# Novel sequences of subgroup J avian leukosis viruses associated with hemangioma in Chinese layer hens

**DOI:** 10.1186/1743-422X-8-552

**Published:** 2011-12-21

**Authors:** Wei Pan, Yulong Gao, Fenfen Sun, Litin Qin, Zaisi Liu, Bingling Yun, Yongqiang Wang, Xiaole Qi, Honglei Gao, Xiaomei Wang

**Affiliations:** 1 Division of Avian Infectious Diseases, State Key Laboratory of Veterinary Biotechnology, Harbin Veterinary Research Institute, Chinese Academy of Agricultural Sciences, Harbin 150001, PR China; 2 Harbin Veterinary Research Institute, 427 Maduan Street, Harbin 150001, PR China

**Keywords:** Avian leukosis virus subgroup J, Layer, Hemangiomas, Sequence analysis, Transcriptional regulatory elements

## Abstract

**Background:**

Avian leukosis virus subgroup J (ALV-J) preferentially induces myeloid leukosis (ML) in meat-type birds. Since 2008, many clinical cases of hemangioma rather than ML have frequently been reported in association with ALV-J infection in Chinese layer flocks.

**Results:**

Three ALV-J strains associated with hemangioma were isolated and their proviral genomic sequences were determined. The three isolates, JL093-1, SD09DP03 and HLJ09MDJ-1, were 7,670, 7,670, and 7,633 nt in length. Their gag and pol genes were well conserved, with identities of 94.5-98.6% and 97.1-99.5%, respectively, with other ALV-J strains at the amino acid level (aa), while the env genes of the three isolates shared a higher aa identity with the env genes of other hemangioma strains than with those of ML strains. Interestingly, two novel 19-bp insertions in the U3 region in the LTR and 5' UTR, most likely derived from other retroviruses, were found in all the three isolates, thereby separately introducing one E2BP binding site in the U3 region in the LTR and RNA polymerase II transcription factor IIB and core promoter motif ten elements in the 5' UTR. Meanwhile, two binding sites in the U3 LTRs of the three isolates for NFAP-1 and AIB REP1 were lost, and a 1-base deletion in the E element of the 3' UTR of JL093-1 and SD09DP03 introduced a binding site for c-Ets-1. In addition to the changes listed above, the rTM of the 3' UTR was deleted in each of the three isolates.

**Conclusion:**

Our study is the first to discovery the coexistence of two novel insertions in the U3 region in the LTR and the 5' UTR of ALV-J associated with hemangioma symptoms, and the transcriptional regulatory elements introduced should be taken into consideration in the occurrence of hemangioma.

## Background

Avian leukosis virus subgroup J (ALV-J), the most recently discovered avian exogenous retrovirus, is thought to have emerged via a recombination event between an unknown exogenous ALV and an endogenous retrovirus [1-3]. Since the first report of the prototype ALV-J strain HPRS-103 in 1989 in the United Kingdom [1,4,5], the virus has been found worldwide, predominantly in meat-type chickens [5]. In China, ALV-J infection of broilers was first detected and officially recognized in 1999 [6], followed by scattered reports of infection of broiler and local chickens in some areas of China [7-9]. Although egg-type chickens have been experimentally infected with ALV-J to induce tumors [10], no field cases of ALV-J infection and tumors in commercial layer chickens were found in China until 2004 [11]. Curiously, ALV-J infection in Chinese layer flocks has become widespread since 2008. Cases of ALV-J, infection and tumors in commercial layer chickens have been emerging in China in recent years, causing severe production problems in layer flocks [12-15].

Clinical infection with ALV-J is associated with the development of various tumors such as myelocytomas (ML), erythroblastosis (EB), hemangiomas, nephromas, and sarcomas [16,17]. ML is the most commonly reported neoplastic syndrome associated with ALV-J in broiler chickens [17]. However, the dominant tumor induced by ALV-J infection in Chinese layer chickens is hemangioma. Large numbers of clinical hemangioma cases in egg-type chickens have been reported in China [7,12-15]. Hemangiomas are vascular tumors characterized by the abnormal growth of endothelial cells from capillary blood vessels [18]. This type of tumor may be induced in chickens by an avian leukosis retrovirus (ALV) variant that contains the erb-B oncogene [19]. It seems that the ALV-Js common in the field induce a greater spectrum of tumors because ML has also occurred in some hemangioma cases [7,14]. Whether the change in the tumor spectrum of ALV-J in chickens is related to the sequence variation of this virus or the species of the host is still unknown.

To better understand the molecular characteristics of ALV-J, three hemangioma isolates, JL093-1, SD09DP03 and HLJ09MDJ-1, isolated from three separate provinces in China, were first confirmed to be associated with ALV-J. Then, the whole genomic sequences of the three isolates were determined and compared with the published sequences of ALV-J strains, including hemangioma and ML strains. Interestingly, two novel 19-bp insertions in the U3 LTR and the 5' UTR, most likely derived from other retroviruses, were found in all the three isolates. These insertions introduced one E2BP binding site in the U3 LTR and RNA polymerase II transcription factor IIB and core promoter motif ten elements in the 5' UTR. Meanwhile, the binding sites for NFAP-1 and AIB REP1 in the U3 LTR of the three isolates were lost, and a 1-base deletion in the E element of the 3' UTR of JL093-1 and SD09DP03 introduced a binding site for c-Ets-1. These transcriptional regulatory elements, known as factors associated with vascular tumors [20-23], were speculated to be related to the development of hemangioma. Herein, we report for the first time the coexistence of two 19 bp insertions in a hemangioma ALV-J.

## Methods

### Clinical samples

In late 2009, suspected cases of ALV-J infection erupted in layer flocks in three main chicken-industry provinces (Jilin, Shandong, Heilongjiang) of China. The levels of egg production were dramatically reduced in the affected flocks. Clinical symptoms included hemorrhages in the skin of the phalanges and feather follicles. Some layer hens had gray-white nodules in the liver, spleen or kidneys, and the liver and spleen were enlarged up to several times their normal size. Morbidity in some flocks reached 60%, and the mortality rate was over 20%, causing heavy economic losses. To confirm and analyze the pathogenesis of this disease, livers, spleens, and tumors were collected from sick chickens in the three provinces.

### Virus isolation

The diseased tissues or tumors were inoculated onto the DF-1 cells, and incubated at 37°C with 5% CO2 for five days for each passage [24]. Uninfected DF-1 cells were used as negative control. After one blind passage, the existence of ALV-J in DF-1 cells was verified by PCR detection of 545 bp repeated sequence [25]. Three blind passage were conducted until the result of PCR detection of cellular genome and RT-PCR detection of supernatant were both positive.

### Primers

Four primers pairs were designed to amplify in the whole genomic sequences of ALV-J isolates (Table 1). Primer pairs W1, W2, and W3 were used to amplify the sequence from position 1 - 7841 of the prototype ALV-J strain HPRS-103, and primer pair W4 was used to amplify the long terminal repeat (LTR) sequences from circularized ALV-J viral DNA.

**Table 1:** Primers for PCR in amplification of cDNA fragments of the three strains

Segments	Primers	Sequences(from 5'- 3')	Product sizes/bp
W1	W1F	GGTGTAGTGTTATGCAATACTC	2782
	W1R	ACAGGCGTGTGGTCTGGCTTCC	
W2	W2F	GGACTGTTGCGCTACATCTGGC	2788
	W2R	GACCCACACGTTTCCTGGTTG	
W3	W3F	GTGCGTGGTTATTATTTCCGTT	2330
	W3R	ACCAATGTGGTGGGAGGTAAA	
W4	W4F	TTAGGAAGGCAACAGACGG	466
	W4R	GGGCGACCAGAATCACG	

Primers W1, W2, W3 used to amplify the sequence from position 1 - 7841 of the prototype ALV-J strain HPRS-103. Primers W4 used to amplify the long terminal repeat (LTR) sequences from circularized viral DNA of the three isolates

### Genomic DNA extract and PCR amplification

The total DNA was extracted from DF-1 cells infected with virus by using a sodium dodecyl sulfate (SDS) - proteinase K and phenol/chloroform/isoamylol (25:24:1) protocol. Genomic DNA PCR amplification was performed according to the manual of rTaq kit (Takara, Dalian, China), by using the proviral genomic DNA as template. The optimum conditions for PCR were as follows: 95°C for 5 min, 30 cycles at 95°C for 30 s, 55°C for 30 s, 72°C for 2 min 40 s, and a final elongation at 72°C for 10 min. The PCR product was analyzed in 1% agarose in Tris-borate - EDTA (TBE) buffer gel containing 0.5 mg/ml ethidium bromide.

### Cloning and sequencing of proviral genomic DNA

All PCR productions were then cloned into the TA vector PMD18-T (TAKARA, Biotechnology Co., Ltd., Dalian, China), and they were sequenced with an AB 3730 DNA sequencer by commercial services in China. Due to the possibility of genetic variation, at least three independent plasmids were sequenced for confirmation.

### Sequence analysis

To analyze the sequences of hemangioma ALV-J isolates, the sequences of reference ALV-J strains, including the sequences of all published ML and other hemangioma ALV-Js, were obtained from GenBank. The accession numbers are listed in Table 2. Multiple sequence alignment was carried out using the sequence analysis software Lasergene 1 (DNASTAR Inc., Madison, WI) and Clustal X 1.83 [26]. An unrooted phylogenetic tree was generated by the distance-based neighbor-joining method using MEGA 4.1 [27]. Bootstrap values were calculated using 500 or 1000 replicates of the alignment. Transcriptional regulatory elements in the U3 were analyzed by NSITE (Recognition of Regulatory motifs), an online service of Soft Berry (http://linux1.softberry.com/berry.phtml).

**Table 2:** ALV-J reference strains and other retroviruses used in this study

Strains	Year	Country	Origin host	Tumor type	Accession No.
HPRS-103^a^	1995	UK	white broiler	ML	Z46390
ADOL-7501^a^	2001	USA	white broiler	ML	AY027920
NX0101^a^	2001	China	white broiler	ML	DQ115805
YZ9902^a^	1999	China	white broiler	ML	HM235670
SD07LK1^a^	2007	China	commercial layer	ML	FJ216405
JS-nt^a^	2003	China	white broiler	ML	HM235667
NHH^a^	2007	China	commercial layer	He	HM235668
SCDY1^a^	2009	China	parental layer	He	HQ425636
JS09GY3^a^	2009	China	commercial layer	He, ML	GU982308
JS09GY6^a^	2009	China	commercial layer	He, ML	GU982310
NM2002-1^a^	2002	China	white broiler	ML	HM235669
HLJ09MDJ-1	2009	China	helan layer	He	JN624878
JL09JL3-1	2009	China	commercial layer	He	JN624879
SD09DP03	2009	China	commercial layer	He	JN624880
JL10HW02^c^	2010	China	commercial layer	He	HQ634801
HLJ10SH04^c^	2009	China	commercial layer	He	HQ634814
HuB09WH02^c^	2009	China	commercial layer	He	HQ634804
HuB09WH03^c^	2010	China	commercial layer	He	HQ634805
HLJ10SH01^c^	2010	China	commercial layer	He	HQ634806
HLJ10SH03^c^	2010	China	commercial layer	He	HQ634813
HLJ10SH02^c^	2010	China	commercial layer	He	HQ634807
HN1001-1^c^	2010	China	commercial layer	He	HQ260974
HN1001-2^c^	2010	China	commercial layer	He	HQ260975
HN1001-3^c^	2010	China	commercial layer	He	HQ260976
PR2257^b^	1989	Czechoslovakia	unknow	sarcoma	L21974
RSV-SRA^b^	1995	USA	unknow	sarcoma	U41731
IC4^b^	1991	France	unknow	L	X77628
RAV-1^b^	1990	France	unknow	L	M62407
RSV-SRB^b^	1998	USA	unknow	sarcoma	AF052428
HBI^b^	1984	Germany	unknow	L	M11784
RAV-1^b^	1990	France	unknow	L	M62407

^a^strains can obtain the whole sequences from GenBank.

^b^strains are other retroviruses published.

^c^strains only env gene available in GenBank.

ML means myeloma leukosis, He represents hemangioma, and L remarks lymphoid tumors

## Results

### Virus isolation and identification

Three ALV-J strains were isolated from the sick chickens, and were designated as JL093-1, SD09DP03, and HLJ09MDJ-1. The genomic DNA of infected DF-1 cells were positive in PCR detection, and the supernatant of infected DF-1 cells was positive in RT-PCR detection (data not shown), indicating the existence of proviral genomic DNA of ALV-J in infected DF-1 cells.

### Sequence comparison of the three isolates and other hemangioma and ML strains

The whole genome sequences of the three isolates were aligned with DNASTAR (version 5.01), and the sequences were submitted to GenBank with the accession numbers as follows: HLJ09MDJ-1 (JN624878), SD09DP03 (JN624880), and JL093-1 (JN624879). The full-length proviral genome sequences of JL093-1, SD09DP03 and HLJ09MDJ-1 were 7670, 7670 and 7633 nt in length, respectively. To better characterize ALV-Js associated with hemangioma on a molecular level, we compared the whole sequences of the three isolates with the sequences of four other hemangioma strains and eight ML strains published in GenBank (Table 2).

### The homology analysis of gag, pol and env genes of the three isolates

Comparisons of the three major genes revealed that the homology of nucleotide sequence of pol and gag genes between the three isolates and other isolates in GenBank were 94.5-98.6%, 97.1-99.5% at the amino acid (aa) level, respectively. These data suggested that the pol and gag genes of all ALV-Js were highly conserved.

**Figure 1:**
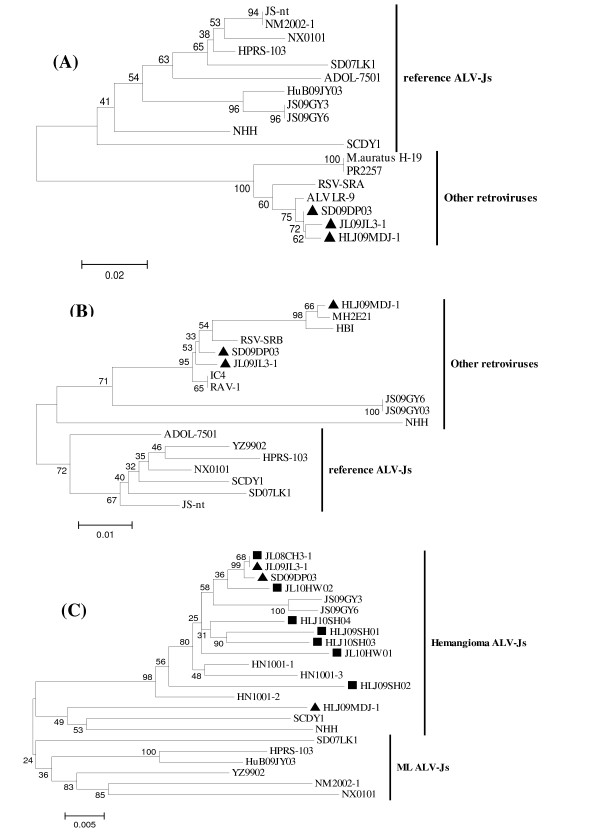
**Phylogenetic relationships of the three isolates and other strains including refenrence ALV-Js and other retroviruses based on U3 5' LTR (Figure 1A), 5' UTR (Figure 1B) and env gene (Figure 1C)**. Isolates marked with a black triangle are three isolates in our study, and strains marked with a black square are also hemangioma ALV-Js from GenBank

However, hemangioma and ML ALV-Js seem to have different env genes. env genes of the three isolates and other hemangioma isolates was 88.5-91.7% identical to that of ML strains at the aa level, but a much higher identity (92.7-99.6%) was found with all hemangioma strains (except for HLJ09MDJ-1 and NHH), which may be indicative of the close relationship among hemangioma isolates. Phylogenetic analysis demonstrated that hemangioma isolates belonged to the same branch and that the reference ML strains clustered in the other branch (Figure 1C).

### Novel sequences in LTRs and 5'UTRs of the three isolates

**Figure 2:**
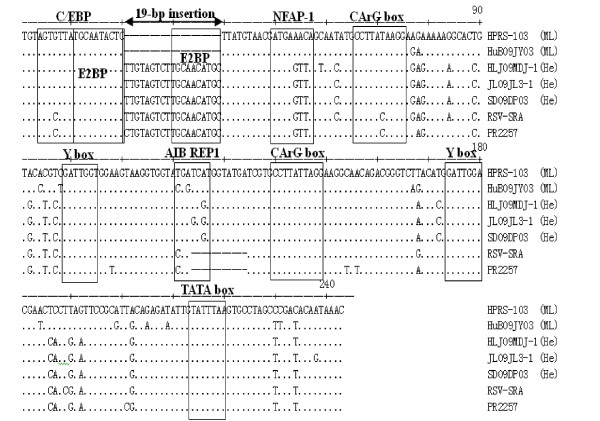
**Comparision of transcriptional regulatory elements in the U3 region of reference strains**. Shown are the sequences of U3 from HPRS-103 [5]. Sequences identical to HPRS-103 are showed as dot (.) and the deletions are represented by dashes (-).The 19 bp novel insertion in the three isolates is marked and transcriptional regulatory elements in U3 area are boxed

A 19-bp insertion (5' CTGTAGTCTTGCAACATGC 3'), located between sites 19 and 20 according to the sequence of the prototype ALV-J strain HPRS-103, occurred in the U3 region in the LTR of the three isolates (Figure 2). This insertion is essentially also identical to sequences found in some other retroviruses, including human hamster virus PR2257 and Rous sarcoma virus strain Schmidt-Ruppin A (RSV-SRA). NSITE, an online service provided by Soft Berry, showed that some transcriptional regulatory elements of the U3 region of the three isolates had changed (Figure 2). In contrast with ML strains, in the three isolates, the NFAP-1 and AIB REP1 components were lost due to the mutation and deletion of base pairs, which was also observed in the hemangioma strain SCDY1 [12]. An E2BP binding site was introduced by the 19 bp-insertion. Other components, including a C/EBP, two CArG boxes, two Y boxes, and one TATA box, were still well conserved. Homology analysis showed that the U3 region in the LTRs of the three isolates shared 91.8-95.3% homology with those of other retroviruses, but only 81.7-87.1% similarity with ML ALV-Js. The U3 sequences of the three isolates clustered in a distinct group apart from homologous sequences, while other hemangioma strains such as SCDY1 and NHH belonged to the same branch as ML strains in the phylogenetic tree (Figure 1A).

**Figure 3:**
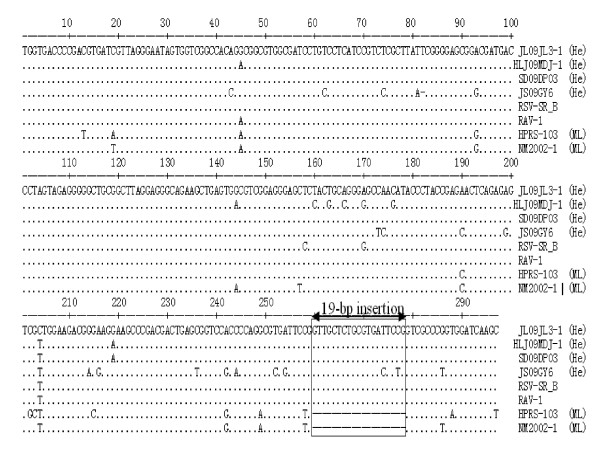
**Phylogenetic analysis of ALV-Js and other retroviruses based on 5' UTR**.

Another 19-bp sequence (5' TGCTCTGCGTGATTCCGGT 3'), located between sites 259 and 260 according to 5' UTR of HPRS-103, was found in the 5' UTRs of the three isolates; this insertion is also present in the hemangioma strain JS09GY6 but is not present in any ML ALV-J strain published (Figure 3). Interestingly, the 19-bp insertion was predicted to introduce RNA polymerase II transcription factor II B and core promoter motif ten elements, which are also present in two hemangioma strains, SCAU-HN06 and JS09GY6 [13,14]. Homology analysis revealed that the 5' UTRs of the three isolates were closely associated with those of type 1 Rouse sarcoma related virus (RAV-1), Rous sarcoma virus strain Schmidt-Ruppin A (RSV-SRB) and other retroviruses, sharing 97.5-100% identity with each other. Phylogenetic analysis also demonstrated that all hemangioma ALV-Js and other retroviruses belonged to the same branch and that, strikingly, the published ML strains were present in the other branch (Figure 1B).

### The molecular features of the 3' UTRs of the three isolates

Three elements, the redundant transmembrane region (rTM), the direct repeat 1 (DR 1) and the E element, are located in the 3' UTR. The molecular characterization of the 3' UTR of the three isolates was as follows.

**Deletion of most of the rTM **Consistent with ML strains, most of the rTMs of the three isolates were deleted, as observed in other hemangioma strains such as SCAU-HN06 and JS09GY6 [13,14] and the ML strain ADOL-7501. The rTM was found only in earlier ML strains such as HPRS-103 [1]. The deletion of the rTM is common in current ALV-J isolates, suggesting that this region might be dispensable for viral fitness [13,14].

**Complete conservation of DR 1 **As a single or double copy element flanking the src in some oncogenic sarcoma viruses, DR 1 had been found exclusively in sarcoma viruses and ALV-J in the past [1]. To our knowledge, DR 1 is present in the 3' UTR of all ALV-J isolates, without any exception. DR 1 may contribute to the fitness of the viruses that contain them because the element is associated with the efficient accumulation of unspliced RNA in the cytoplasm and the selective increase in the amount of spliced src mRNA in ALV.

**Figure 4:**
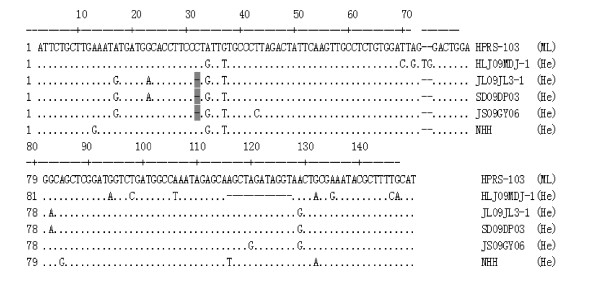
**Sequence comparison of E element of hemangioma ALV-Js with the prototype ML ALV-J strain HPRS-103**. 1-bp deletion was coloured by grey background

**A binding site for c-Ets-1 was introduced in the E element **Most base pairs of the E element were conserved in the three hemangioma isolates, but minor mutations were observed (Figure 4). For example, the E elements of JL093-1 and SD09DP03 had a 1-base deletion (between 7387 and 7389 according to the HPRS-103 sequence); this deletion was also present in JS09GY6, JS09GY3, JS09GY5 and SCAU-HN06 [13,14]. The deletion introduced a specific distinct binding site for c-Ets-1 according motif analysis; this binding protein is associated with vascular endothelial cell differentiation. In contrast, most parts of the E element of ML strains whose sequences have been reported to date have been deleted. For example, in the USA, the E element of ML strains have been reported to have substantial deletions in >50% of ALV-Js [28], and the E element has been completely deleted in earlier Chinese ML isolates such as YZ9902 and NX0101 and the recent strain JS-nt.

## Discussion

In this study, the whole genomic sequences of three ALV-J isolates associated with hemangioma from layers were determined and compared with the published sequences of ML and hemangioma ALV-J strains. Although the three isolates were collected from different sick layers in three separate provinces of China, they all contained special sequences such as modifications of the LTRs and 5' UTRs that seem to be molecular markers of some hemangioma strains.

Consistent with other ALV-J strains, gag and pol genes of the three isolates were highly conserved. However, env genes of hemangioma strains seem to differentiate from ML strains, since the fact that all hemangioma strains were clustered in the same branch while ML strains belonged to the other branch in the phylogenetic tree (Figure 1C). The env glycoprotein of ALV, as in other retroviruses, functions mainly as a ligand for receptor binding for virus entry into susceptible cells [29], and was also demonstrated to be a major determinant of the lineage-specific oncogenicity and host range [30-32]. Whether the tumor spectrum is determined by the env genes of ALV-J or not needs further study.

Hemangioma ALV-Js seem to contain special U3 region in the LTR and 5' UTR sequences. Phylogenetic analyses based on the U3 LTR and the 5' UTR revealed that all hemangioma isolates examined and some other retroviruses belonged to the same branch, whereas the published ML ALV-J sequences belonged to another branch (Figure 1A, 1B), implying that these U3 LTR and the 5' UTR sequences of hemangioma ALV-Js were most likely derived from some other retroviruses. To our knowledge, the ALV-J strains reported to date have been recognized as slowly transforming viruses, while related retroviruses such as HBI and RSVs quickly induce tumors in chickens and have altered biological properties [33,34]. For example, HBI, which was derived from the myc-containing virus MC29, was demonstrated to be capable of inducing a variety of tumors when injected into newborn chickens [33]. Hence, the acute-like sequences of those hemangioma strains may influence the tumor spectrum and pathogenicity of ALV-J in layers. The three isolates in this study can replicate to a high titer in DF-1 cells, a characteristic that is shared with some other hemangioma viruses [29]. This new biological property may have a close relationship with the U3 of the 5' LTR and the 5' UTR contained in the genome of ALV-Js.

It is noteworthy that there were two 19 bp insertions in the U3 LTR and 5' UTR in our three isolates that have not been previously reported. The U3 LTR region of the avian retroviruses has been extensively characterized as a model of a strong transcription regulatory unit. This compact enhancer and promoter drives high levels of viral and cellular gene transcription in many cell types in birds and in mammals [35-37]. According to our analysis, parts of the transcriptional regulatory elements in the U3 region of the three isolates have changed. The binding sites for NFAP-1 and AIB REP1 were not observed in our three isolates but exist in all ML ALV-Js. The NFAP-1 site is recognized by activator protein 1 (AP-1). AP-1 is a heterodimeric protein that regulates gene expression in response to stimuli such as cytokines, growth factors, stress, and bacterial and viral infections [20]. AIB REP1, known as repair of chromatin damage 1, was recently reported to be a regulatory factor of human gene Apo-AI. Apo-AI is closely related to the vascular invasion of hepatocellular carcinoma in humans [21]. The absence of the NFAP-1 and AIB REP1 binding sites may influence the infection abilities of the ALV-J virus in chickens or the development of hemangioma. Moreover, the insertion in the U3 introduced an E2BP binding site. E2BP was first found in hepatitis B virus (HBV) and influences the liver specificity of HBV replication through the combination with an enhancer, EII [22]. The additional DNA binding site for E2BP introduced in the U3 may be related to viral tropism [23]. The insertion or deletion of transcription regulatory elements may severely impact the transcriptional activity of the LTR in hemangioma isolates, thereby affecting the replication or infection capability of this virus.

The 5' UTR played an important role in viral replication through potential intra- and intermolecular interactions [38]. One RNA polymerase II transcription factorIIB element and core promoter motif ten elements were introduced via a 19-bp insertion in the 5' UTR of all three isolates; this insertion is also found in the hemangioma strains SCAU-HN06 and JS09GY6 [13,14]. However, the influence of the inserted sequence in viral replication is still unknown.

Elements in the 3' UTR play a key role in virion assembly and the ability to induce tumors [39]. Early studies found that the rTM exists in the majority ALV-J trains in China; however, deletion of the rTM has been observed in a large number of ALV-J strains in recent years [13,14,40]. The loss of the rTM seems to be a trend in the sequence variation of the 3' UTR in current ALV-Js in China (unpublished data). Therefore, the rTM is suspected to be related to the evolution and virulence of this virus, and its role should not be ignored. The E element, containing a biding site for the transcription factor c/EBP and acting as an enhancer, was previously only found in the gene of sarcoma viruses [41]. Currently, the E element is present in many ALV-J strains in addition to our isolates (unpublished data). The existence of the E element in the 3'UTR does increase the occurrence rate of tumors in chickens infected with ALV-J, although this element is not the decisive factor in the induction of tumors [42]. For the hemangioma ALV-Js examined, a binding site for c-Ets-1, introduced in JL093-1 and SD09DP03 due to a 1-bp deletion, was associated with the development of hemangiomas. Consistent with other reports, DR 1 of all hemangioma ALV-Js was very well conserved. This high level of conservation is consistent with the fact that DR 1 plays an important role in the assembly of the genomic RNA of ALV-Js [43].

In conclusion, our study is the first to discovery the coexistence of two novel insertions in the U3 region in the LTR and the 5' UTR of ALV-J associated with hemangioma symptoms, and the transcriptional regulatory elements introduced should be taken into consideration in the occurrence of hemangioma.

## Competing interests

The authors declare that they have no competing interests.

## Authors' contributions

WP carried out the molecular genetic studies, participated in the sequence alignment and drafted the manuscript. YLG guided the study design, and revised the manuscript. FFS, LTQ, BLY, ZSL, YQW, XLQ, HLG helped in experiments. XMW participated in its design and coordination and helped to draft the manuscript. All authors read and approved the final manuscript.
